# Treatment of the depressive patients at clinical high-risk for psychosis

**DOI:** 10.1192/j.eurpsy.2022.691

**Published:** 2022-09-01

**Authors:** M. Omelchenko

**Affiliations:** Mental Health Research Centre, Youth Psychiatry, Moscow, Russian Federation

**Keywords:** early intervention, antidepressant, Clinical high-risk, antipsychotic

## Abstract

**Introduction:**

At present, there is no universally approach to treating patients at clinical high-risk for psychosis (CHR) without comorbid mental disorders. However, if there are revealed depressive symptoms, proper treatment becomes necessary.

**Objectives:**

Establish pharmacological classes and doses of drugs that have proved effective in treating depressive patients at CHR.

**Methods:**

A comparative study of pharmacological classes and doses of drugs was carried out, showing the effectiveness in treatment of 219 depressive patients at CHR and 52 depressive patients without CHR. The treatment effectiveness was carried out on the reduction of depression symptoms on the HDRS scale, and the CHR symptoms on the SOPS scale.

**Results:**

A significant reduction of depression symptoms was achieved in the group of depressive patients with and without CHR on the HDRS scale (67.9% and 76.6% respectively). The reductions of the CHR symptoms were 46.1% and 53.3% respectively. There were differences between the severity of depression symptoms and CHR symptoms before and after the treatment. Both groups used antidepressants followed by the prescription of antipsychotics to increase the effectiveness of the therapy. No difference was found in the doses of antidepressants for the fluoxetine equivalent (46.0 vs 42.6 mg per day, p 0.05) and some differences were found for the average effective doses of antipsychotics for the chlorpromazine equivalent (385.4 vs 230.8 mg per day, p 0.05).

**Conclusions:**

The same pharmacological classes are used for the treatment of young depressive patients with and without CHR, but the former have significantly higher doses of antipsychotics.

**Disclosure:**

No significant relationships.

